# Different perspectives of prison guards and mental health workers in forensic care

**DOI:** 10.3389/fpsyg.2024.1420565

**Published:** 2024-08-27

**Authors:** Jasvant Timar, Evi Buurman, Koen Westen, Philippe Delespaul

**Affiliations:** ^1^Reinier van Arkel, s-Hertogenbosch, Netherlands; ^2^GGZ Breburg, Breda, Netherlands; ^3^Avans University of Applied Sciences, Breda, Netherlands; ^4^CCAF, Utrecht, Netherlands; ^5^Department of Psychiatry and Neuropsychology, South Limburg Mental Health Research and Teaching Network, EURON, Maastricht University, Maastricht, Netherlands; ^6^Mondriaan ggz, Heerlen, Netherlands

**Keywords:** treatment perspectives, forensic care, policy dynamics, collaborative interactions, communication challenges

## Abstract

**Objectives:**

This study investigates the differences in treatment perspectives of prison guards and mental health practitioners within a Psychiatric Prison Unit (PPU).

**Methods:**

This qualitative study uses questionnaires and focus groups to explore the relationships between prison guards (*N* = 4) and mental health professionals (*N* = 6) working at the Psychiatric Prison Unit in Zwolle, the Netherlands. Two questionnaires (the Recovery Attitude Questionnaire and the Recovery Knowledge Inventory) were completed by the participants. A selected subsample based on diverging beliefs concerning treatment perspectives was recruited for additional focus groups. The dialogues were transcribed and coded into a comprehensive scheme. Responses were analyzed to describe perceptions and attitudes of respondents towards forensic care.

**Results:**

This study identified three main themes: policy, communication, and person-dependent factors. Understaffing and high turnover rates in the correctional facility led to prioritization of safety concerns over treatment objectives. Guards and mental health professionals had different communication styles which hindered the alignment of treatment goals. Person-dependent factors, including personality traits and individual attributes, were significant in shaping collaborative interactions.

**Conclusion:**

This study reveals agreement in the viewpoints between prison guards and mental health professionals but highlights the complex challenges in providing effective treatment within the confines of a correctional facility. These challenges are influenced by policy dynamics, communication limitations, and individual-specific factors.

## 1 Introduction

The aim of mental health care within correctional settings is to reduce recidivism through optimal patient rehabilitation (Custodial Institutions Agency, 2022). Several studies have indicated that recidivism of criminal behavior is partly due to the presence of a psychiatric disorder (Vermeiren et al., 2000; Benda et al., 2001). It has been suggested that mental illness serves as a catalyst for criminal involvement and, consequently, that treatment of the mental illness is the remedy (Skeem et al., 2014). The highest prevalence among incarcerated individuals was for inmates with psychosis spectrum disorders (Baranyi et al., 2019). Empirical evidence indicates that inmates with psychosis spectrum disorders have an increased likelihood of recidivism in comparison to non-afflicted inmates (Fazel and Yu, 2011; Igoumenou et al., 2015). The higher recidivism rates for this subgroup emphasizes the need for better treatment of high-risk mentally ill inmates (Igoumenou et al., 2015).

In the Netherlands, care for inmates with health-related problems spans from regular health care to specialized forensic psychiatric treatment (Van Marle, 2007). Some inmates have severe mental health issues that require specialized treatment. When an individual’s mental health renders them at risk in the conventional prison milieu, the alternative is placement within a Psychiatric Prison Unit (PPU). In a PPU, inmates live in smaller groups, with more structure and protective measures. For instance, smaller groups allow for increased focus on each inmate’s specific needs. Providing more individualized attention to inmates results in several benefits, including the capacity for more intensive therapeutic approaches. Additionally, it becomes easier for staff to monitor progress and intervene promptly when necessary. Offering more structure can also lead to greater predictability, which in turn contributes to a calmer environment.

Targeted interventions are typically administered by an interdisciplinary team of psychologists, psychiatrists, and nurses. These clinicians use motivational interviewing and different behavioral strategies (Lamberti, 2007; Igoumenou et al., 2015). Within the PPU context, prison guards are trained to identify signs of mental and/or psychological distress. Moreover, they are taught to adapt their communication styles during interactions with mentally ill detainees, in line with the principles of respectful engagement (Custodial Institutions Agency, 2022). Motivational interviewing is a therapeutic approach designed to encourage inmates with psychiatric conditions to engage in treatment and make positive behavioral changes. It is accomplished by applying empathy, individualized goal setting and focusing on collaboration.

During incarceration, people have time and can take the opportunity to seek treatment for psychosis spectrum disorders (Lamberti, 2007; Igoumenou et al., 2015). Also, in prison people can find refuge and access to rudimentary amenities, including more balanced nutrition and specialized care (Abram et al., 2003; Van Marle, 2007). The collaborative partnership between prison guards and mental health professionals is essential to create this therapeutic environment (Day et al., 2012). The primary goals of the prison staff are safety, ensuring the humane treatment of offenders, and inmate reintegration. Research studying the influence of prison staff on prison conditions often include demographic predictors such as gender, age, years of service, or tenure (Cheeseman et al., 2001; Hemmens and Stohr, 2001). Studies devote less attention to factors such as staff attitudes towards inmates or treatment methodologies (Molleman and Leeuw, 2012). The perception of the prison environment by prison staff is important to understand the prison context.

Differences are evident among prison staff in relation to the core treatment goals within the correctional system (Tewksbury and Mustaine, 2008). Important factors include sex, education, and experience; these shape the perspectives, attitudes, and actions of prison staff. Female and college-educated staff attribute higher importance to rehabilitation in comparison to their male and less-educated counterparts. In comparison, male staff and those without college education prioritize retribution and incapacitation. The nature of one’s job role defines the perspectives, attitudes, and behaviors of prison staff with regards to treatment paradigms. For instance, security staff operating within prison units tend to perceive prisons as retribution-oriented environments, where punitive measures are often invoked to regain control (Schaftenaar and Helm Van Der, 2014). As staff members accumulate experience, there is a gradual shift from the belief that criminal offenders are incarcerated primarily for punishment and deterrence. Instead, there is an increasing recognition that the purpose of imprisonment should focus on education and rehabilitation (Tewksbury and Mustaine, 2008).

An exploration of the viewpoints, attitudes, and behaviors of (mental) health practitioners operating within a prison setting is lacking. One study (Watson et al., 2004) found that nurses functioning within a prison environment are insecure in their role. Conflict between the “divergent aims” of correctional officers and nurses arose due to the inherent “underlying assumptions” that underpin health care provision and correctional functions.

The mental health care domain perceives correctional staff often as unduly severe and punitive (Appelbaum et al., 2001). For example by over-reliance on physical restraints or force to control inmates. This is likely due to cultural differences. The correctional culture typically involves regimentation, universally applied rules, the implicit authority of security staff, and punitive sanctions for inmate violations. In contrast, the culture of the health professions is characterized by individualized treatment, informed consent, and negotiated compliance. Correctional staff perceive mental health providers therefore as excessively lenient, susceptible, and overly indulgent towards inmates because they are highly adaptable to their patients’ needs. Sauter et al. (2019) demonstrated in their study that collaboration between these professional cultures can create a more therapeutic environment within correctional settings, enabling transformation of these spaces into therapeutic communities. The study found that improved communication and shared objectives between correctional staff and health providers led to reduced use of punitive measures and increased focus on rehabilitation and individualized care. As a result, inmates reported feeling more supported and respected, with noted improvements in their mental health and engagement in rehabilitative programs. Correctional staff and health providers also reported positive changes in their perceptions of each other, highlighting the value of a collaborative approach in enhancing inmate outcomes. The result is a therapeutic environment that embraces the principles of a “positive” or “open” living environment, characterized by employee support, adaptive conduct, focused teaching of coping strategies to patients, and minimizing punitive measures (Schaftenaar and Helm Van Der, 2014). This leads to increased patient treatment motivation, favorable treatment outcomes, heightened empathy, and reduced criminal behaviors. Conversely, a “closed” living environment, characterized by limited employee support, limited prospects for patient growth, a strict environment, and recourse to repression and punishment can lead to reduced patient treatment motivation, aggressive behaviors, emotional instability, exacerbated psychological issues, and ultimately, less favorable treatment outcomes.

The present study attempts to investigate the potential differences in the treatment perspectives of prison guards and mental health practitioners (psychologists, psychiatrists, and nurses) operating within a Psychiatric Prison Unit (PPU).

## 2 Materials and methods

### 2.1 Study design

This study was conducted using qualitative research methods. As the number of professionals was limited, multiple focus groups were not feasible and therefore saturation is defined by inclusion of the entire population of informants. The consolidated criteria for reporting qualitative studies (COREQ) was used as a framework for conducting and reporting our study (Tong et al., 2007).

### 2.2 Participants and recruitment

This study was conducted within the Psychiatric Prison Unit in Zwolle, the Netherlands. Two distinct focus groups were conducted as part of this study: one with prison guards and another with mental health professionals (including psychologists, psychiatrists, and nurse specialists). The recruitment process involved enlisting both prison guards and mental health professionals employed within the respective unit. Therefore, a convenience sample was used.

In focus groups where different professional groups are integrated, there is a risk that participants may exhibit reluctance in expressing their opinions due to the dominant hierarchical position of other participants. Therefore, the decision was made to segregate the prison guards and the mental health workers into distinct groups.

Inclusion criteria involved participants having proficient command of the Dutch language to meaningfully participate in group discussions. Involvement in this study was entirely voluntary and predicated upon informed consent. No type of remuneration or incentives were provided to prevent potential biases in participation.

At the start of each focus group session, an explanation of the study’s objectives and guidelines regarding confidentiality was given to all participants. Every group member explicitly agreed to the recording of audio during the sessions. All the collected material and data were subjected to thorough anonymization protocols.

### 2.3 Procedure

Adverts were disseminated within the units. Interested individuals received two documents: the information letter and an informed consent form. After receiving consent, participants were given two questionnaires related to staff’s attitudes toward recovery; the Recovery Attitude Questionnaire and the Recovery Knowledge Inventory. The information was used to select subjects for the respective focus groups. The guiding principle in this selection process was to include participants within each group who held divergent opinions or perspectives concerning treatment. The questionnaires were not utilized any further, as they were deemed beyond the scope of this study.

Preceding each focus group, a comprehensive overview of the project was provided to all participants, underscoring the commitment to confidentiality. Each session lasted for approximately 80–90 min. After the focus groups, a debriefing session among the researchers was conducted to explore outcomes and to identify avenues for further exploration. Both focus group discussions were centered around three primary inquiries: elucidation of one’s contribution to public safety, how is the collaboration between prison guards and therapists experienced, and the exploration of avenues for improvement. The direction and facilitation of these groups was orchestrated by JT. JT conducted the focus groups, documented the discussions, presented additional inquiries, and monitored interactions among the participants.

### 2.4 Data analysis

The focus groups were audio-recorded to facilitate comprehensive documentation. These recorded dialogues were subsequently transcribed verbatim, with meticulous attention to ensure full anonymity. The transcriptions were then inputted into Atlas.ti, a software tool used for qualitative data analysis, to facilitate an inductive qualitative content analysis. Atlas.ti was utilized to manage, extract, and analyze patterns, themes, and insights within the transcripts. This analytical process involved the application of codes, categories, and themes, and was underpinned by an inductive qualitative content analysis framework aligned with the Generic Qualitative Approach (Hsieh and Shannon, 2005; Braun and Clarke, 2006). This approach ensures flexibility and a focus on the meanings derived from the participants’ experiences, allowing themes to emerge naturally from the data. To ensure the accuracy of the codes and categories, two researchers (JT and EB) independently coded the data. The findings were subjected to rigorous deliberation within the research team (JT, EB, KW, and PD). A comprehensive coding scheme was subsequently formulated, followed by a thorough examination and discussion of the interrelationships between categories and themes.

## 3 Results

**Figure 1:**
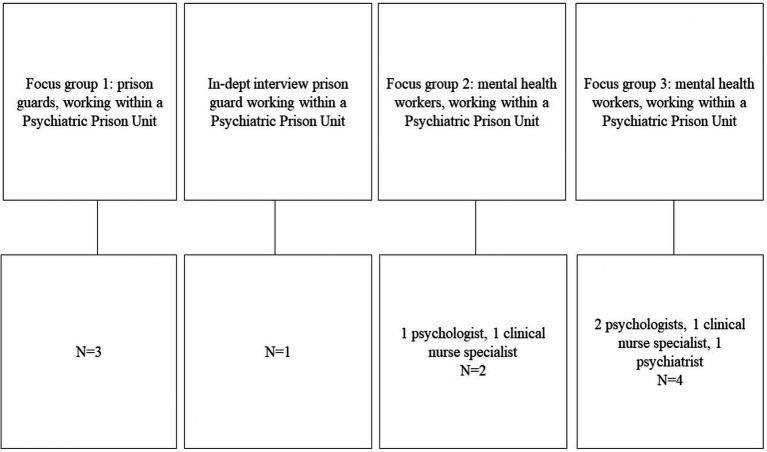
Overview of focus groups and in-depth interview.

Figure 1 provides an overview of the participants involved in the focus groups. Unfortunately, due to organizational challenges, not all eligible participants were able to participate in the scheduled focus groups. In total, 3 focus groups with 2–4 participants, were conducted: one for the guards, and two for the clinicians. One intended focus group, where only one participant appeared, was transformed into an in-depth interview.

### 3.1 Theme 1: policy

The first theme relates to policies. An important concern is the scarceness of personnel. This results in current employees feeling overworked which impacts the efficacy of the provided treatments. This was affirmed by both prison guards and mental health practitioners. Within the Psychiatric Prison Unit (PPU), there are multiple complex requirements that contribute to a safe environment. These complex requirements can make incidents more likely, which may lead to escalating security measures. Given the lack of personnel, the team faces increased demands, leading to an inadequate allocation of time for the prioritization of treatment-oriented endeavors. The irregular presence of mental health professionals and the absence of a unified framework for their coordination (i.e., therapist actions are limited to their specialization) further emphasizes these challenges. Adding to this complexity, the PPU is comprised of different units, each with its own idiosyncrasies.

The respondents acknowledge that the prison climate stabilizes the inmates. The problem emerges after stabilization. Transitioning acquired skills to extramural life remains problematic. Staff members feel stifled by constraints imposed by a prison system that predominately focus on security. However, changing the prison environment is a slow process which faces many challenges.

#### 3.1.1 Quotes

##### 3.1.1.1 Prison guards

- *‘For example, the staff shortage is so great that we cannot offer an evening program. Well, that’s just embarrassing, is not it? It’s not in anyone’s interest that we, a Psychiatric Prison Unit, are not open in the evenings. Look how long those people are staying behind closed doors.’*
- *‘No, I try not to get involved with other units. I mean, I have my own unit. And as long as it’s going well there, that’s what I care about. I have a certain unit where I would rather not work. So sometimes I ask my colleagues if they want to work there instead of me. Sometimes it works, sometimes it does not.’*

##### 3.1.1.2 Mental health workers

- *‘I’m really enjoying my job, because there’s a lot that can be done, and I find the population really fun and interesting. But it’s also very complicated, because you have to navigate between care and security all the time. In detention, you cannot do everything to provide the right care, so you always have to make sacrifices. That can be difficult. You have to think about what is possible and what is good care.’*
- *‘And if there is another incident, the interests are closer to security and control. Look at a recent incident at xxx (a different prison penitentiary in the country where one of the guards was killed). I was already afraid that everything would be tightened up here, but it’s not too bad. That is of course the reflex, in these types of institutes. When something serious happens, draconian measures come from The Hague.’*

### 3.2 Theme 2: communication

The second theme distilled from the focus groups and in-depth interview is the lack of effective communication. Prison guards mentioned these shortcomings, emphasizing problems related to coordination. Their work occurs in the patient environment, and they shared that when the mental health workers from the treatment team access the unit, they seldom communicate with them. The guards stressed that their insights, deliberations, and observations are not effectively incorporated. One recurring complaint was that it is difficult to get consultation for specific patients, often due to the unavailability of certain specialists who have different work schedules.

The mental health workers underscored the significance of patient-centric communication and typically thought this task to be the guards’ role. The mental health workers expected the guards to be responsible for fostering a therapeutic climate within the unit. This includes effective communication with patients, which was assumed to be crucial. The treatment team argued that multidisciplinary meetings frequently lacked adequate preparation on the part of the guards. These meetings, they argued, are crucial for planning and coordinating treatment. They felt that the guards occasionally lack clarity regarding the treatment objectives for patients. As such, the treatment team were also aware of the problems regarding communication, as evidenced by their observations regarding treatment objectives.

#### 3.2.1 Quotes

##### 3.2.1.1 Prison guards

- *‘No, some people come on to the unit, walk into a room, have a conversation, and then leave. You do not know how that person is feeling. In fact, go and ask for prior information, it might be helpful to have a prison guard sit in during the conversation with that client. Recently a psychiatrist who just walked on the unit was assaulted. Yes, you know, that brings so much risk with it. Be aware of where you work. Come and consult us before you go into a conversation.’*
- *‘That is sometimes the case… it first needs to escalate before steps can be taken. We all know that person, we know how the situation will continue to develop. We could be one step ahead, but that is not possible. So, then you are basically just waiting for it to escalate, even though as prison guards you can give so many signals. But it takes a while before you are heard. That can be very frustrating sometimes. You can always voice your opinion and if a different decision is made in the end: yes of course you let them know. You tell them you do not agree with this.’*

##### 3.2.1.2 Mental health workers

- *‘No: it has to be both. Safety is not only in the isolation cells but is especially important in proximity, knowledge and contact. And that is also what sometimes seeps onto the unit. The treatment of people and that you do not immediately say: if you do this again, I will put you back in the cell. Or someone who is suicidal, goes straight to the isolation cell. Those are all reflexes. But you really have a lot less of that here. So, the treatment here is 99% of the time fine. That is something that we all work on together.’*
- *‘Very briefly: a patient is working for an hour with a therapist to regulate his aggression. But then you have many other situations where that focus is perhaps a little less, while events can still occur on the unit that have to do with aggression regulation. This is when it is difficult to translate the skills: oh, now you are angry, what have you learned during the session to deal with this? This may be a simple example, but this is the core. But what I also find important is that they know what the goals of the treatment are and that they report them. I know that on some units, they always make working points out of this, which I would also like to do, but I think they are a bit averse to it here. They do not want to do it.’*

### 3.3 Theme 3: person-dependent

The third theme is the “person-dependent” dimension. Prison guards state that the interaction with the treatment team is related to individual attributes. As a group, they experience a spectrum of emotions ranging from adversarial to affirmative. They value mental health professionals who are consistently present, which does not happen frequently because most mental health professionals operate on irregular schedules. Furthermore, the distinctive personality of each therapist defines the nature of their collaboration. Differences between mental health professionals are evident in the extent to which they share treatment-relevant information with the guards. Some are open, others are more reserved. The guards prefer to engage with mental health professionals who are accessible, credible, and inclined to consider the guards’ perspectives.

Reciprocally, the mental health workers experience individual differences in dealing with prison guards. Some guards proactively assumed leadership roles and strived to collaborate with the broader team in patient treatment. In contrast, there are guards who prioritized safety and are reluctant to engage in more risk-taking approaches within the treatment context. There is also a subset of guards that is susceptible to the influence of narratives related to specific patients. The mental health professionals further observed inter-personal differences in psychiatric awareness between guards.

#### 3.3.1 Quotes

##### 3.3.1.1 Prison guards

- *‘With one therapist, you know: okay, that therapist is empathetic and they take action, and you think it’s going to be okay. With the other, you have something like: well, that therapist is probably going to wait and see, and if possible, they’ll pass it on to someone else. Often you do not have a choice, and that is the only therapist available. Then you have to work with that therapist, regardless of the relationship you have with them. And I can imagine that if you have a poorer relationship with that therapist, you start to wonder if you should consult this therapist. Knowing that they may say or do this. But you do not want to think like that.’*
- *‘Some mental health professionals have such a busy schedule, they come in and you do not really get anything out of it. Some do not give any feedback. They just quickly say; ‘yes, it went well. I have to go to the next one.’ While another one says; ‘we’ll just sit down.’ And with those you can have a decent conversation. One time someone literally said: “yes, that’s none of your business. I am a psychiatrist, I determine my treatments and if something changes, you’ll see it.’*

##### 3.3.1.2 Mental health workers

- *‘There are also people who are very safety-oriented and others who are very care-oriented. I still have the impression that the people who are more safety-oriented are also a little less involved in the treatment. And yet it is very nice that they are there, because they also point out our blind spots. This is also very important in forensic psychiatry.’*
- *‘In the past, prison guards had a lot of difficulty with antisocial borderline women who showed a lot of physical violence. At some point, it was said: “Okay, they have to move on to a calmer, more group-appropriate climate.” So, we would like to sign her up for the mixed unit. At that time, stories about that woman would go around: “she did this and that, super antisocial and blah blah. Everyone hears that and thinks that woman does not belong on the mixed unit at all. There is the fear of disrupting the whole group if such a person arrives. But ultimately, in most cases, it goes: “yes, this woman has to come to the mixed unit, because this is part of her treatment.” I think this is sometimes difficult for some guards to accept. They feel like they are not being listened to.’*

## 4 Discussion

**Figure 2:**
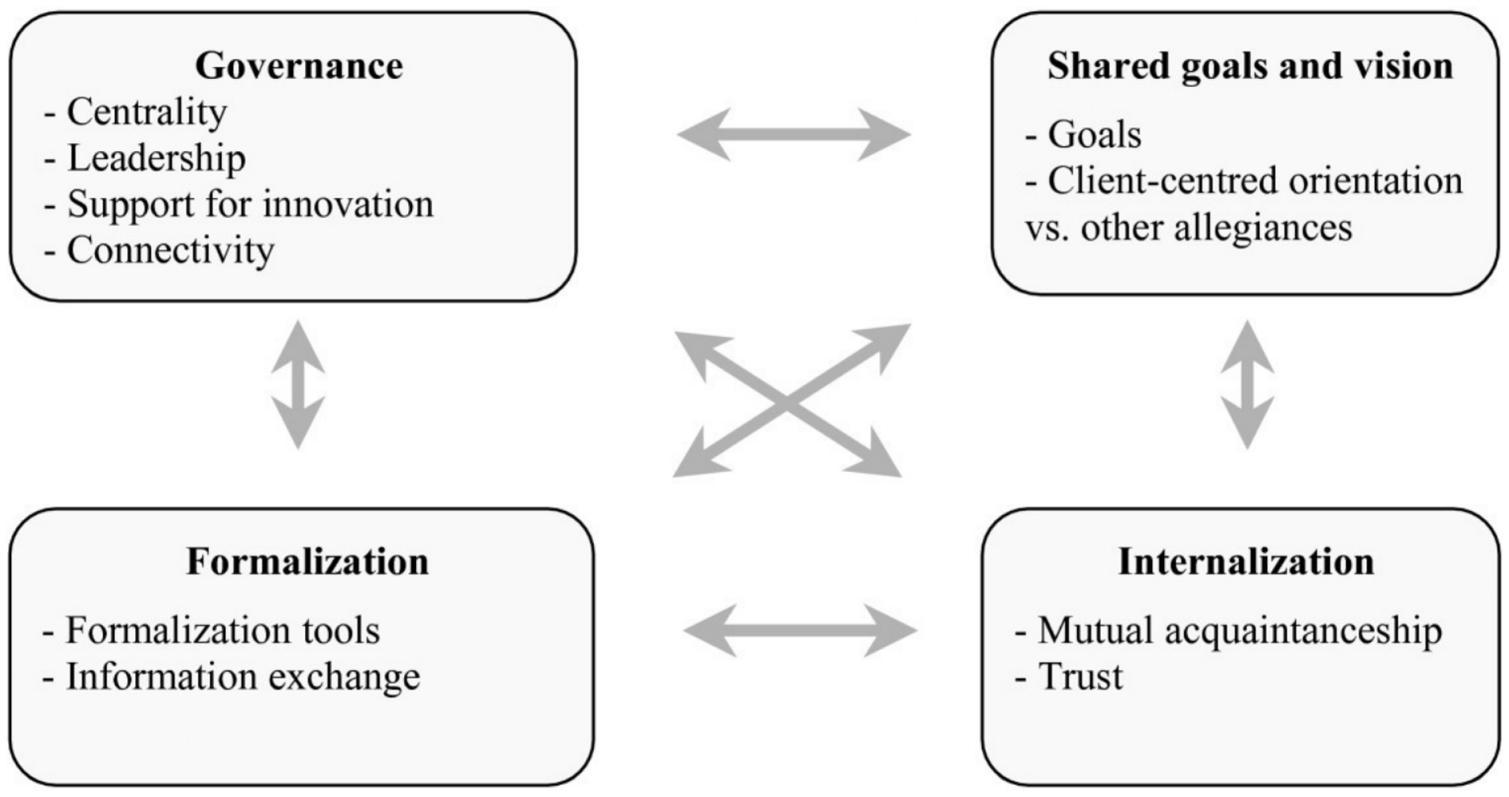
Four dimensional model of collaboration (D’Amour et al., 2008).

**Figure 3:**
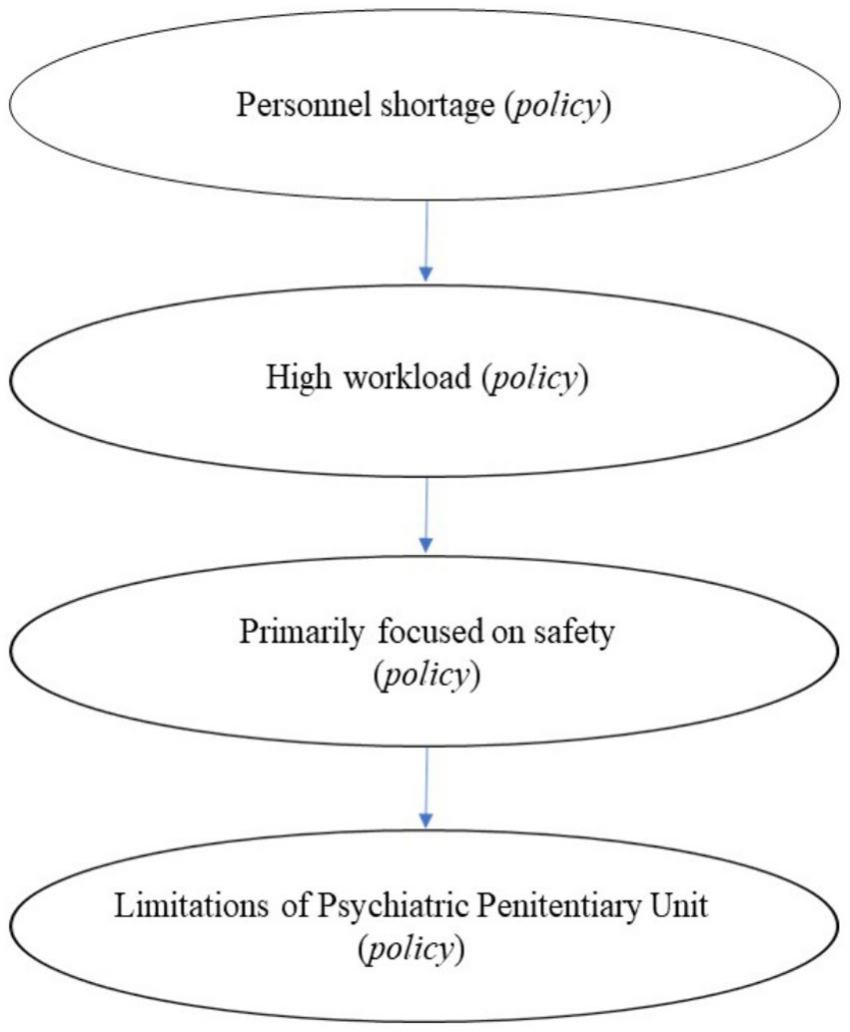
Effects of policy.

This study explored the perspectives of prison guards and mental health workers within a Psychiatric Prison Unit (PPU). The findings highlight agreements in some areas, but also intricate challenges in delivering effective treatment within the constraints of a correctional facility. The dynamics of policy, communication, and person-dependent factors interplay within this context. The Four Dimensional Model of Collaboration (D’Amour et al., 2008) suggests that effective collaboration is multifaceted (Figure 2). Governance, a central element within the model, highlights the significant impact of policies on collaborative efforts. Figure 3 illustrates the influence of policy on treatment processes within the unit. However, these policies impose limitations on the available communication channels and individual-specific factors.

Restrictions arising from prison policies make it difficult to align the treatment visions between guards and mental health professionals. Understaffing and high turnover rates are central to this challenge. This leads to a cascade of effects. The scarcity of staff increases the workload for existing personnel, particularly with care-intensive patients prone to aggression. Consequently, guards prioritize safety, and this can overshadow other considerations, such as investment in a therapeutic relationship. This is congruent with the findings of Lambert et al. (2012), who described challenges associated with the establishment of a therapeutic alliance in situations that contain dual roles. The context of a PPU potentially creates tension between the objectives and visions of mental health professionals, with a primary focus on treatment, and guards who are in charge of safety. Additionally, the distinct operational environment of a PPU limits the therapeutic options available through penal codes, regulations, and directives.

**Figure 4:**
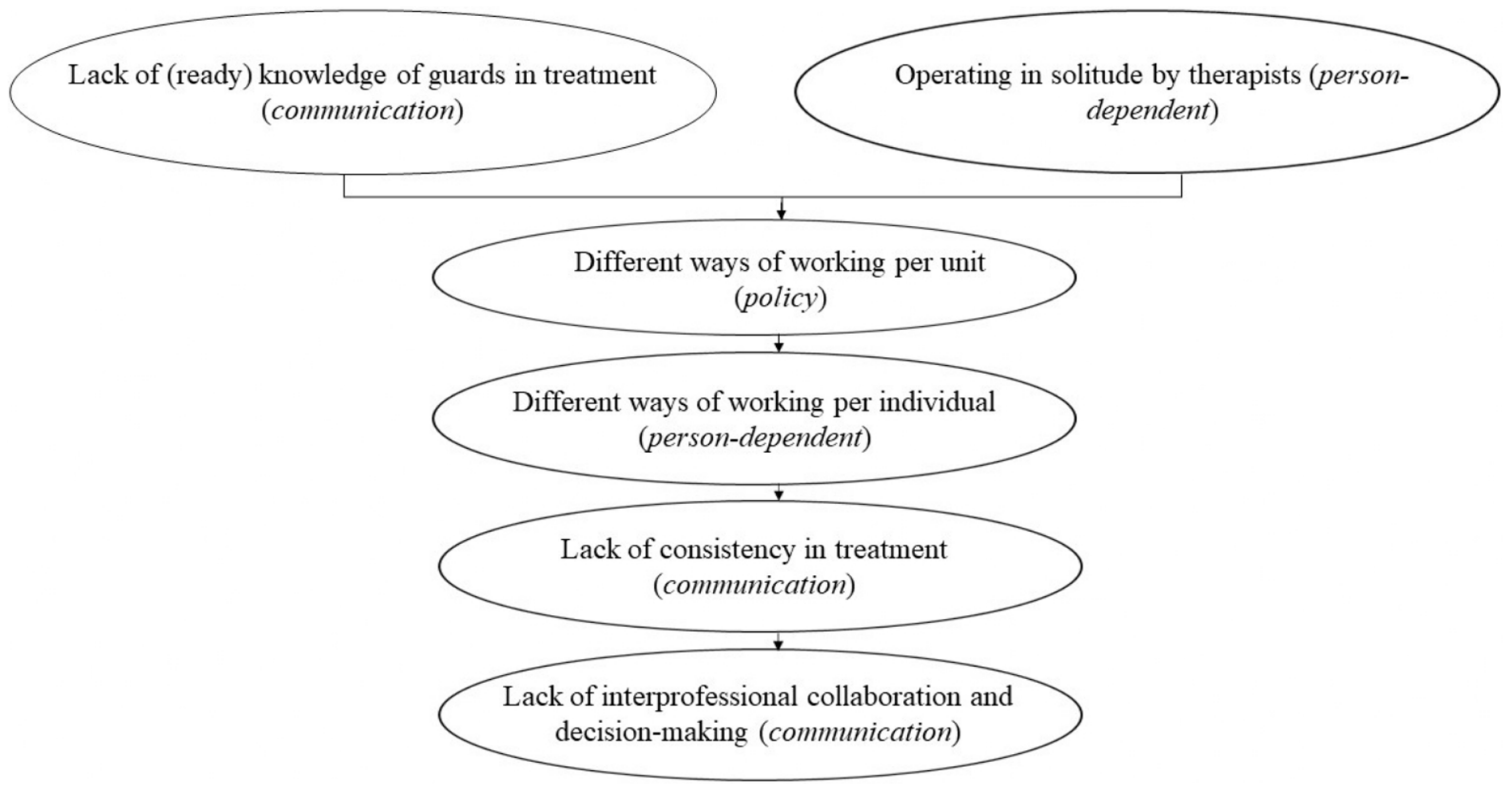
Effects of communication.

Nevertheless, prison guards agreed that an increased emphasis on treatment is necessary. According to the guards, achieving this objective requires greater coordination between them and the treatment team. The effects of communication on the other thematic elements are summarized in Figure 4. The mental health professionals emphasized that their role is to shape the treatment environment within the unit in collaboration with the guards. This necessitates an awareness of treatment objectives and involvement in treatment-related meetings. Both mental health professionals and guards acknowledge the absence of cohesion within the prison setting. Different units adopt distinct operational approaches, resulting in a state of disorder and ambiguity in staff schedules on the unit. Moreover, this also necessitates further knowledge on mental health. Given the varying psychiatric conditions, distinct approaches are required.

The guards perceived differences in the willingness to collaborate from the mental health professionals. They noted discrepancies in therapist engagement; the most involved mental health professionals seek more coordination. Both guards and mental health professionals observed significant individual variances. Having shared goals and visions is a crucial factor for ensuring effective collaboration (Day et al., 2012; Minkman, 2017). Minkman emphasized that a unified approach and mutual understanding among team members are essential for successful interprofessional collaboration, as it ensures all team members are working towards the same objectives. Both prison guards and mental health professionals need to develop and align their goals to prioritize both safety and therapeutic outcomes. This alignment can help bridge the gap between the security-focused approach of the guards and the treatment-focused approach of the mental health professionals.

The present study found that both parties wanted to contribute to the effective treatment of patients yet felt they were limited by governance policies. In addition, problems also exist in the internalization of roles. Both parties do not appear to be sufficiently aware of the interdependencies. The current findings highlight the absence of an immediate sense of cohesion within the PPU. Problems occur when there is an inadequate exchange of information. This results in difficulties in establishing an effective treatment-focused collaboration.

The effect of the staff shortage results in higher (unrealistic) expectations on the guards. This is consistent with Mitchell et al. (2011) findings that staff shortages lead to increased workload and stress, which can undermine collaborative efforts and the overall effectiveness of treatment. However, the guards and mental health professionals both recognize that they need to work together. The current study shows that the guards wish to contribute more to treatment, but that this is not feasible due to scheduling constraints. Additionally, work on the Psychiatric Prison Unit is dynamic, and flexibility is required in response to events. Mental health professionals do their best to consider the needs of the team, but they have limited time to coordinate with everyone. As such, it is not realistic to involve everyone in decision-making when working in a high-pressure environment. Within the realm of possibilities, both parties have indicated that it is important to improve communication. This requires a willingness to engage in meetings. The guards felt that more coordination with them could take place and that their expertise should be considered in decision-making. The mental health professionals suggested that, for instance, the multidisciplinary meetings could be better prepared.

In conclusion, this study integrates with previous research on interprofessional collaboration, particularly in constrained environments. The challenges identified—stemming from policy restrictions, communication barriers, and role dualities—are consistent with broader findings in the literature. Addressing these issues through improved governance, communication, and education could enhance the collaborative process and ultimately improve treatment outcomes within correctional facilities.

## 5 Research limitations

One limitation of this study is the sample size of only 10 participants from the same facility. Participation was strictly voluntary. As such, it is possible that the individuals who did choose to enlist in the study have a predisposition to engage in collaborative discussions, particularly to create change. This phenomenon underscores the theme of person-dependency. Individuals who did not participate potentially have contrasting viewpoints and may be less inclined to articulate them openly. Therefore, it is advisable to design strategies to incorporate individuals holding divergent perspectives for future research.

Another limitation of this study was the role of the primary researcher (JT). He conducted the focus groups, documented the discussions, presented additional inquiries, and monitored interactions among participants. Focus groups are ideally conducted by two people. Consequently, the consideration of non-verbal communication in the present study was potentially limited. Future research should take this into account.

It is also important to incorporate the patient’s perspective into subsequent research endeavors. This relates to how patients perceive the collaboration between correctional facility guards and mental health professionals, as well as an assessment of the influence of these dynamics on their treatment experiences and outcomes. To get a more comprehensive overview, it can be important to longitudinally investigate the effects of staff shortages and high turnover rates on treatment outcomes.

Additionally, it would be beneficial to conduct a comparative analysis with other similar Psychiatric Prisons Units (PPUs). Such a comparative approach could elucidate whether the issues identified in this study are location specific. By delving deeper into these research avenues, a more comprehensive understanding of the intricate aspects surrounding the provision of treatment within correctional facilities can be attained. This, in turn, may lead to enhancements in patient care and the facilitation of more effective collaboration among diverse healthcare professionals.

## Data Availability

The raw data supporting the conclusions of this article will be made available by the authors, without undue reservation.
